# The clinical diagnostic value of plasma miR-592 and miR-217-3p levels in retinoblastoma

**DOI:** 10.5937/jomb0-34794

**Published:** 2022-10-15

**Authors:** Luo Jin Yan, Huang Bin Lin, Hu Qi Yu, Li Ru Jie, Jun Chen, Yuan Ling Mei, Yuan Peng

**Affiliations:** 1 Nanchang Hongdu Hospital of Traditional Chinese Medicine, The Five Senses of Chinese Medicine, Nanchang City, China; 2 Jiangxi University of Traditional Chinese Medicine, The Five Senses of Chinese Medicine, Nanchang City, China; 3 Affiliated Hospital of Jiangxi University of Traditional Chinese Medicine, The Five Senses of Chinese Medicine, Nanchang City, China

**Keywords:** retinoblastoma, miR-592, miR-217-3p, correlation, retinoblastom, miR-592, miR-217-3p, korelacija

## Abstract

**Background:**

This study was designed to investigate the abnormal expression of plasma miR-592 and miR-217-3p in retinoblastoma (Rb) and explore the clinical diagnostic value of their expression levels for Rb.

**Methods:**

The 100 Rb patients who came to Nanchang Hongdu Hospital of Traditional Chinese Medicine from January 2018 to January 2019 were selected as the Rb group, and 100 healthy patients who came to the physical examination centre during the same period were selected as the control group. Real-time fluorescence quantitative PCR (qRT-PCR) was used to detect the expression levels of plasma miR-592 and miR-217-3p in all subjects; analyse the relationship between plasma miR-592 and miR-217-3p levels and the clinicopathological characteristics of Rb. Pearson correlation analysis evaluated the relationship between plasma miR-592 and miR-217-3p levels and overall survival.

**Results:**

Plasma levels of miR-592 and miR-217-3p in the Rb group were significantly higher than those in the control group (p<0.0001), and the expression of miR-592 was significantly correlated with family genetic history (p 0.0001), tumour bias (p=0.0081), lymph node metastasis (p=0.0048) and pathological grade (p=0.0025), and the expression of miR-217-3p was significantly related to family genetic history (p 0.0001), optic nerve infiltration (p 0.0001), lymph node metastasis (p=0.0090), and pathological grade (p 0.0001). The high expression of miR-592 and miR-217-3p presents a more serious pathological manifestation of Rb, and the overall survival of patients is significantly shortened with the increase of miR-592 (r=-0.2276, p=0.0052) and miR-217-3p levels (r=-0.6461, p 0.0001).

**Conclusions:**

and miR-217-3p are highly expressed in the plasma of Rb patients, and their elevated levels present severe pathological manifestations of Rb and shortened overall survival, which is expected to become biomarkers for clinical diagnosis of Rb.

## Abstract (sr)

**Uvod:**

Cilj ove studije je bio da ispita patološku ekspresiju miR-592 i miR-217-3p u plazmi kod retinoblastoma (Rb) i istraži kliničko-dijagnostičku vrednost nivoa njihove ekspresije za Rb.

**Metode:**

Izabrano je 100 pacijenata koji boluju od Rb i koji su do{li u bolnicu tradicionalne kineske medicine Nanchang Hongdu od januara 2018. do januara 2019. godine kao Rb grupa, a 100 zdravih pacijenata koji su došli u centar za fizikalni medicinu u istom periodu je odabrano kao kontrolna grupa. Za detekciju nivoa ekspresije miR-592 i miR-217-3p u plazmi kod svih osoba je korišćen kvantitativni PCR test sa fluorescencijom u realnom vremenu (kRTPCR) kao i za analizu odnosa između nivoa miR-592 i miR217-3p u plazmi i kliničko-patoloških karakteristika Rb. Za procenu odnosa između nivoa miR-592 i miR-217-3p u plazmi i ukupnog preživljavanja, koriššćena je Pirsonova korelaciona analiza.

**Rezultati:**

Nivoi miR-592 i miR-217-3p u plazmi u Rb grupi bili su značajno viši od onih u kontrolnoj grupi (p < 0,0001), a ekspresija miR-592 je bila značajno povezana sa porodičnom genetskom istorijom (p < 0,0001), prisutnošću tumora (p = 0,0081), metastazama u limfnim čvorovima (p = 0,0048) i stepenom patoloških promena (p = 0,0025), dok je ekspresija miR-217-3p bila značajno povezana sa porodičnom genetskom istorijom (p < 0,0001), infiltracijom optičkog nerva (p < 0,0001), metastazama u limfnim čvorovima (p = 0,0090) i patološkim stepenom (p < 0,0001). Visoka ekspresija miR-592 i miR217-3p predstavlja ozbiljniju patološku manifestaciju Rb, a ukupno preživljavanje pacijenata je značajno skraćeno sa povećanjem miR-592 (r = -0,2276, p = 0,0052) i miR - 217-3p nivoa (r = -0,6461, p < 0,0001).

**Zaključak:**

miR-592 i miR-217-3p su visoko izraženi u plazmi pacijenata sa Rb, a njihovi povićeni nivoi predstavljaju teške patološke manifestacije Rb i ukazuju na kraće ukupno preživljavanje. Očekuje se da miR-592 i miR-217-3p postanu biomarkeri za klinčku dijagnozu Rb.

## Introduction

In children, retinoblastoma (Rb) is a relatively common human genetic malignant ocular tumour [1] [2] [3] [4]. It has the characteristics of high incidence, and low survival rate usually occurs in children under 3 years old and can affect one or both two eyes and seriously affect the health of children [5] [6] [7]. Rb can be divided into two genetic modes: germline inheritance and non-germline inheritance [8]. Among them, germline inheritance is dominated by germ cell pathogenic variants, manifested as bilateral Rb (about 20-30%) or unilateral multiple Rb (about 70-80%), while somatic pathogenic variants cause nongermline inheritance, mainly manifested as unilateral Rb [9] [10] [11]. At present, the diagnosis of Rb mainly relies on the experience of ophthalmologists through ophthalmoscopes, ultrasound, computed tomography (CT), and magnetic resonance imaging (MRI) [12] [13]. Due to the delay in diagnosis and treatment, and there is still no objective hematological biomarker differential diagnosis, most Rb patients only come to the hospital with symptoms such as white pupils or strabismus, and the prognosis is often poor [14]. At present, the pathogenesis of Rb has not been fully elucidated. Therefore, early diagnosis and standardized treatment of Rb has always been a hot spot in ophthalmic tumour research. The key factor of objective blood markers for early diagnosis may improve Rb patients' eye salvage rate and survival rate [15].

MircoRNAs (miRNAs) are short non-coding RNAs (about 22 nucleotides) in cells [16] [17]. They can degrade or block mRNA translation by binding to the complementary sequence of the 3'-end non-coding region of target mRNAs, thereby regulating the various biological processes such as cell proliferation, differentiation, and apoptosis [18] [19]. It has been shown that circulating miRNA can stably exist in various body fluids, such as blood, urine, and saliva. Compared with other diagnostic methods, circulating miRNAs are non-invasive and obtain faster results [20]. More and more studies have shown that miRNA expression can change various cellular pathogenesis signals and may become a new biomarker for tumour diagnosis and a potential target for tumour treatment [21] [22] [23]. It has been found that various tumours and cancer cells, e.g., prostate cancer and osteosarcoma, secrete miR-217, which can be used as a diagnostic marker for the early detection of tumours [24]. Meanwhile, the presence of miR-592 in serum has been correlated with the early detection of colorectal cancer and glioblastoma [25] [26] [27]. However, there are still few studies on miRNA in Rb, and the expression of miR-592 and miR-217-3p in the plasma of Rb patients and their relationship with the clinicopathological characteristics of Rb are still unclear. Based on this, this study detects the levels of miR-592 and miR-217-3p in the plasma of Rb patients, analyses the relationship between the two and the clinical characteristics of Rb, and explores the diagnostic value of the two, providing references for clinical diagnosis of Rb and assessment of the patients' condition.

## Materials and methods

### General information

A total of 100 Rb patients who were admitted to Nanchang Hongdu Hospital of Traditional Chinese Medicine from January 2018 to January 2019 were selected as the Rb group, and the diagnostic criteria refer to the relevant criteria in the Rb international classification system (international intraocular retinoblastoma classification, IIRC) (28). Inclusion criteria: (1) Meet the diagnostic criteria of Rb; (2) According to surgical pathology and imaging examination, the clinical manifestation was diagnosed as Rb; (3) Complete physiological and pathological examination data; (4) Patients and their families signed informed consent. Exclusion criteria: (1) Those with severe malnutrition; (2) Combined with liver, gallbladder, and kidney disease; (3) Combined with autoimmune system disease; (4) Combined with mental illness and family history of mental illness. In addition, 100 healthy people who had undergone physical examination at the physical examination centre of thisNanchang Hongdu Hospital of Traditional Chinese Medicine during the same period were selected as the control group. This study was conducted following the Declaration of Helsinki, in line with medical ethics regulations, and reviewed and approved by the ethics committee of this Nanchang Hongdu Hospital of Traditional Chinese Medicine.

### Clinical data and sample collection

Collect general information of all subjects, including gender and age. According to the International Classification of Retinoblastoma (ICRB), the family genetic history of Rb patients (with or without), tumor bias (unilateral or bilateral), optic nerve infiltration (yes or no), and lymph node metastasis (yes or no) are counted. Whether the tumour cells are arranged in Flexner-Winterstein rosettes, differentiate between well-differentiated and poorly-differentiated. Blood samples of all subjects were collected on an empty stomach in the early morning for subsequent miR-592 and miR-217-3p expression levels.

### qRT-PCR detection of plasma miR-592 and miR-217-3p expression levels

The blood samples were centrifuged at 4000 r/min for 10 min within 2 h after extracting plasma. MiRNeasy Serum/Plasma kit (Qiagen, Hilden, Germany) extracted total RNA from the sample. According to the manufacturer's instructions, the total RNA of the sample was reverse transcribed into cDNA concerning the reverse transcription kit and cDNA synthesis kit. Configure the reaction system according to the SYBR Green qPCR Master Mix kit, and perform qRT-PCR amplification to detect the expression levels of miR-592 and miR-217-3p. U6 was used as an internal reference gene. All primers are designed by GenePharma (Shanghai, China). The primer sequence is as follows: miR-592 forward: 5’-TTGTGTCAATATGCGATGATGT-3’, miR-592 reverse: 5’-GCGAGCACAGAATTAATAGCAC-3’; miR-217-3p forward: 5’-ACAGGCCGGGACAAGTGCAATA-3’, miR-217-3p reverse: 5’-GCTGTCAACGATACGCTACGTAACG-3’; U6 forward: 5’-CTCGCTTCGGGC-AGCACA-3’, U6 reverse 5’-AACGCTTTCACGAA-TTTGCGT-3’.

### Overall survival statistics

Regular follow-up of all Rb patients, once a month for the first 3 months after the end of treatment, once every 3 months if there is no recurrence or progression, once every 6 months if there is no abnormality for 2 consecutive times, continuous 2 times without exception, changed to once a year. The longest follow-up cut-off time is 30 months. By the end of the follow-up, there is no disease progression or death, and the censored data is included in the analysis.

### Statistical analysis

All data were statistically analysed using SPSS 22.0 software (SPSS, Inc., Chicago, IL, USA). The count data were expressed as N (%), and the chisquare test was performed. The measurement data was expressed as the mean ± standard deviation and performed the t-test. GraphPad 8.0 software (GraphPad Software Inc., La Jolla, CA, USA) was used for graphing. *p* <0.05 is considered a statistically significant difference.

## Results

### General clinical data

A total of 100 children with Rb participated in this study. Among all Rb patients, 44 were males, and 56 were females; ages 7 months to 5 years, average (29.80±17.49) months; 52 cases had family genetic history, 48 cases had no family genetic history; 58 cases of the monocular disease, binocular disease 42 cases; 63 cases of optic nerve infiltration, 37 cases of optic nerve infiltration; 49 cases of lymph node metastasis, 51 cases of lymph node metastasis; 48 cases of differentiated cases, 52 cases of undifferentiated cases (Table 1). In addition, 100 healthy children who had physical examinations during the same period served as the control group. There were 53 males and 47 females; they were 6 months to 5 years old, with an average of (28.66±13.75) months. There was no statistical difference in gender and age between the two groups (*p*>0.05), and they were comparable.

**Table 1:** General clinical data of Rb patients.

Category	N (%)
Gender	
male	44 (44.0%)
female	56 (56.0%)
Age	
0–12 months	36 (36.0%)
>12 months	64 (64.0%)
12–36 months	25 (25.0%)
36–60 months	39 (39.0%)
Family history	
Yes	52 (52.0%)
No	48 (48.0%)
Tumour bias	
Monocular disease	58 (58.0%)
Binocular disease	42 (42.0%)
Whether the optic nerve is infiltrated	
Yes	63 (63.0%)
No	37 (37.0%)
Whether lymph node metastasis	
Yes	49 (49.0%)
No	51 (51.0%)
Pathological grade	
Well differentiated	48 (48.0%)
Poorly differentiated	52 (52.0%)

### Comparison of plasma miR-592 and miR-217-3p levels

**Figure 1:**
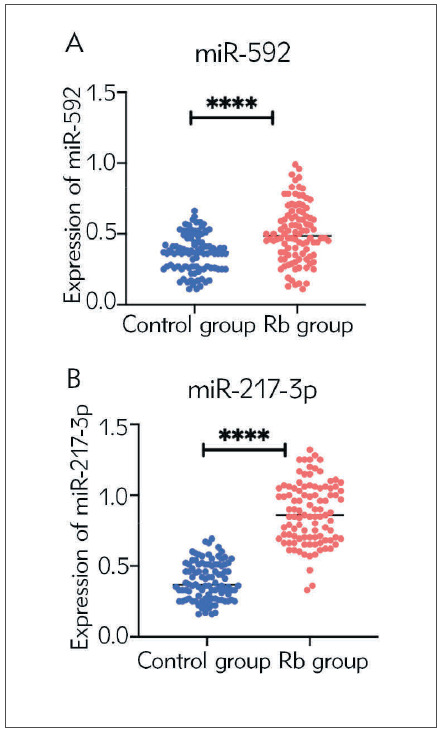
Comparison of plasma levels of miR-592 and miR-217-3p in subjects. (A) Comparison of plasma miR-592 levels in subjects; (B) Comparison of plasma miR-217-3p levels in subjects. ^****^p＜0.0001.

To study the expression patterns of miR-592 and miR-217-3p in Rb, we tested the plasma levels of miR-592 and miR-217-3p in all children tested. The results showed that the Rb group showed higher expression of plasma miR-592 (*p*<0.0001, Figure 1A) and miR-217-3p (*p*<0.0001, Figure 1B) levels than the control group.

### The relationship between miR-592 and miR-217-3p and Rb clinicopathological characteristics

In order to explore the clinical significance of plasma miR-592 and miR-217-3p levels in Rb patients, all Rb patients were divided into low expression groups and high expression groups based on the median expression miR-592 (0.485) and miR-217-3p (0.86), respectively. Then, we analysed the relationship between the expression of miR-592 and miR-217-3p and the clinicopathological characteristics of Rb patients and found that the expression of miR-592 was significantly correlated with family genetic history (*p* 0.0001), tumour bias (*p*=0.0081), lymph node metastasis (*p*=0.0048) and pathological grade (*p*=0.0025), but not related to the patient's gender, age, and optic nerve infiltration, suggesting that the high expression of miR-592 presents more serious pathological manifestations of Rb (Table 2); while the expression of miR-217-3p was significantly related to family genetic history (*p* 0.0001), optic nerve infiltration (*p* 0.0001), lymph node metastasis (*p*=0.0090), and pathological grade (*p* 0.0001), but there is no correlation between the patient's gender, age, and tumour laterality, suggesting that the high expression of miR-217-3p also presents more serious pathological manifestations of Rb (Table 3).

**Table 2:** The relationship between miR-592 and Rb clinicopathological characteristics. ^**^ *p* 0.01, ^****^ *p* 0.0001

Category	miR-592<br>low<br>expression<br>(n=50)	miR-592<br>high<br>expression<br>(n=50)	*p* value
Gender			0.3138
male	25	19	
female	25	31	
Age			0.5323
0–12 months	16	20	
12 months	34	30	
12–36 months	16	9	
36–60 months	18	21	
Family history			<0.0001***
Yes	15	37	
No	35	13	
Tumour bias			0.0081**
Monocular disease	36	22	
Binocular disease	14	28	
Whether the optic<br>nerve is infiltrated			0.2137
Yes	28	35	
No	22	15	
Whether lymph node<br>metastasis			0.0048**
Yes	17	32	
No	33	18	
Pathological grade			0.0025**
Well-differentiated	16	32	
Poorly differentiated	34	18	

**Table 3:** The relationship between miR-217-3p and Rb clinicopathological characteristics. ^**^ *p* 0.01, ^****^ *p* 0.0001.

Category	miR-217-3 low	miR-217-3	*p* value
Gender			0.2266
male	19	25	
female	32	24	
Age			0.6777
0–12 months	17	19	
>12 months	34	30	
12–36 months	13	12	
36–60 months	21	18	
Family history			<0.0001****
Yes	11	41	
No	40	8	
Tumour bias			0.5493
Monocular<br>disease	28	30	
Binocular<br>disease	23	19	
Whether the<br>optic nerve is<br>infiltrated			<0.0001****
Yes	19	44	
No	32	5	
Whether<br>lymph node<br>metastasis			0.0090**
Yes	18	31	
No	33	18	
Pathological<br>grade			<0.0001****
Well-<br>differentiated	14	34	
Poorly<br>differentiated	37	15	

### Analysis of the relationship between the levels of miR-592 and miR-217-3p and the overall survival of Rb patients

**Figure 2:**
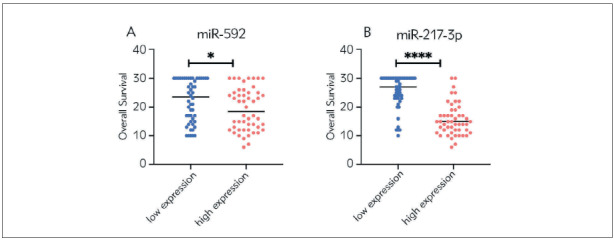
Comparison of overall survival of Rb patients. (A) Comparison of overall survival between low and high expression of plasma miR-592 in patients with Rb; (B) Comparison of overall survival between low and high expression of plasma miR-217-3p in Rb patients. ^*^p<0.05, ^****^p<0.0001.

**Figure 3:**
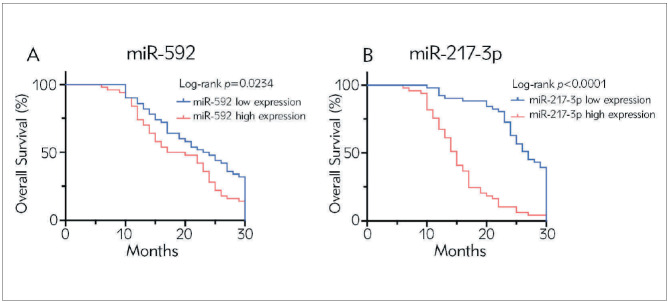
Overall survival with relatively high and low expression of miR-592 and miR-217-3p in Rb patients. (A) Overall survival with relatively high and low expression of miR-592 in Rb patients; (B) Overall survival with relatively high and low expression of miR-217-3p in Rb patients.

**Figure 4:**
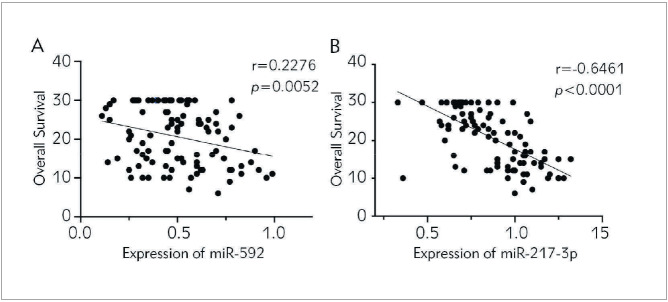
Correlation analysis between the levels of miR-592 and miR-217-3p and overall survival in Rb patients. (A) Correlation analysis between miR-592 level and overall survival in Rb patients; (B) Correlation analysis between miR-217-3p level and overall survival in Rb patients.

In order to evaluate the prognostic value of miR-592 and miR-217-3p levels in Rb patients, we analysed the relationship between miR-592 and miR-217-3p levels and OS. We found that the OS of Rb patients in the miR-592 and miR-217-3p high expression group was significantly shorter than that in the miR-592 (*p* 0.05, Figure 2A; *p* 0.05, Figure 3A), and miR-217-3p (*p* 0.0001 Figure 2B; *p* 0.0001, Figure 3B) low expression group. The Pearson correlation analysis was further carried out. The results showed that the levels of miR-592 and miR-217-3p were negatively correlated with the overall survival of Rb patients. As the levels of miR-592 and miR-217-3p increased, the overall survival of the patient is shortened (miR-592: r=-0.2276, p=0.0052; miR-217-3p: r=-0.6461, p<0.0001; Figure 4A, and Figure 4B).

## Discussion

Rb is a kind of ocular malignant tumour with a family genetic predisposition and mostly occurs in infants under 3 years old [28] [29] [30]. The main clinical symptoms are intraocular hypertension, vitreous turbidity, corneal edema, intraconjunctival hyperemia and edema, and strabismus [31] [32] [33]. In addition, Rb can cause intracranial and distant metastasis, which can easily cause the death of Rb patients, posing a great threat to the quality of life of patients and their offspring [34] [35]. According to statistics, about 9,000 newborns are diagnosed with Rb each year, and about 3,000 children die of Rb [36] [37]. Therefore, finding biomarkers for early diagnosis of Rb is of great significance to improve the diagnosis and treatment of Rb and the prognosis of children [38]. This study aims to evaluate the abnormal expression of plasma miR-592 and miR-217-3p in Rb patients and explore the clinical diagnostic value of their expression levels for Rb.

In recent years, miRNA has been an important biomarker for diagnosing different diseases [39] [40]. miRNAs play an important regulatory role in various biological processes, such as embryonic development and organ formation [41]. In the process of tumorigenesis, miRNA can be used as an effective biomarker for early tumour diagnosis and prognostic evaluation [42]. For example, the expression of miR-204 in Rb patients is dysregulated, and its expression level is related to optic nerve infiltration, lymph node metastasis, and tumour tissue differentiation. miR-204 may affect the proliferation and apoptosis of Rb cells, thereby affecting the development of Rb, which may become an early stage molecular indicator for diagnosing and evaluating the prognosis of Rb [43] [44] [45]; miR-338-5p is up-regulated in the serum of patients with Rb, which may play a role in promoting cancer in the occurrence and development of Rb, and has the potential value of the early diagnosis of Rb [46] [47]. Recent studies have shown that dysregulated miR-592 and miR-217-3p have been reported to be involved in developing many different types of cancer. For example, the high expression of miR-592 is closely related to the tumorigenesis and poor prognosis of colorectal cancer. miR-592 exhibits carcinogenic effects on prostate cancer cells by inhibiting Forkhead box O3A [48] [49]. miR-592 promotes the proliferation, migration, prognosis, and invasion of cancer cells [50] [51]. The expression of miR-217-3p is elevated in metastatic liver cancer tissues and highly aggressive liver cancer cell lines [52] [53]. The high expression of miR-217-3p in thyroid cancer tissues and cell lines is related to the clinical stage and lymph node metastasis of patients. Therefore, it is speculated that miR-592 and miR-217-3p may serve as oncogenic markers for different types of cancer. In this study, we first detected the expression levels of plasma miR-592 and miR-217-3p in Rb patients and found the plasma levels of miR-592 and miR-217-3p in Rb patients were higher than those in the control group, suggesting that the expression level of Rb in children increases. Similar to our studies, it was previously found that elevated serum miR-592 may be tumour-derived. It can differentiate patients with early-stage colorectal cancer and advanced adenoma from healthy individuals. Meanwhile, miR-217 family is considered to have an important role in cancer progression due to its abnormal expression in various tumour tissues. miR-217 in osteosarcoma is higher than the tissues near osteosarcoma, and can affect the prognosis of patients. The expression level of miR-217 is related to the pathological grade and clinical stage of the tumour [24]. The expression levels of miR-592 and miR-217-3p are respectively related to family genetic history, tumour bias, optic nerve infiltration, lymph node metastasis, and degree of differentiation in Rb patients, further suggesting that the levels of miR-592 and miR-217-3p are related to the clinical features of Rb patients. This present study also urges that serum miR-592 and miR-217-3p may be used to differentiate patients at early stages of Rb. The pathological characteristics are closely related. It is speculated that miR-592 and miR-217-3p may play a role in promoting cancer in Rb. We also explored the relationship between the expression levels of miR-592 and miR-217-3p and the overall survival of Rb. As the levels of miR-592 and miR-217-3p increase, the overall survival of Rb patients shortens.

## Conclusion

The expression levels of miR-592 and miR-217-3p in the plasma of Rb patients are significantly increased. They are related to family genetic history, tumour bias, optic nerve infiltration, lymph node metastasis, and degree of differentiation. The increased expression of plasma miR-592 and miR-217-3p will shorten the overall survival of Rb patients. Plasma miR-592 and miR-217-3p may be used as biomarkers for Rb diagnosis and prognosis and even effective targets for treating the disease.

## Dodatak

### Acknowledgments

Not applicable.

### Funding Statements

National Natural Science Foundation of China Youth Science Fund Project. (No. 821049352)

Natural Science Foundation of Jiangxi Province. (No. 20202BAB2060743.)

Science and Technology Research Project of Jiangxi Provincial Department of Education. (No. GJJ190647)

### Conflict of interest statement

All the authors declare that they have no conflict of interest in this work.
